# Supplementary material for: Fibre-specific mitochondrial protein abundance is linked to resting and post-training mitochondrial content in the muscle of men
Source: Nat Commun. 2024 Sep 3;15:7677. doi: 10.1038/s41467-024-50632-2 (PMC11371815; doi:10.1038/s41467-024-50632-2)
Supplement: Supplementary file 1 — Supplementary Information [file 41467_2024_50632_MOESM1_ESM.pdf]

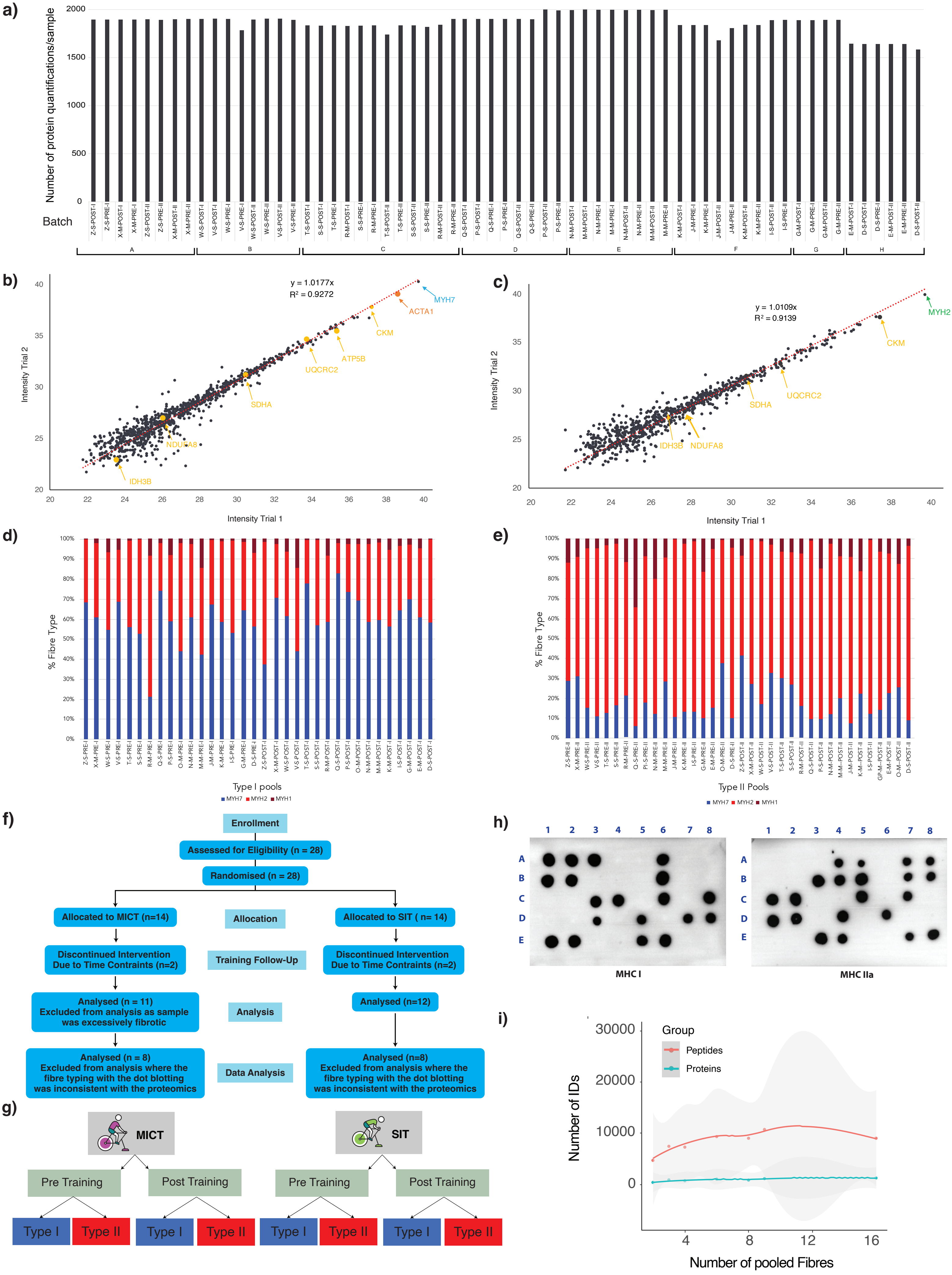
a)
Number of protein quantifications/sample
2000
1500
1000
500
0
Batch
A
B
C
D
E
F
G
H
b)
y = 1.0177x
R² = 0.9272
MYH7
ACTA1
CKM
ATP5B
UQCRC2
SDHA
NDUFA8
IDH3B
Intensity Trial 2
Intensity Trial 1
c)
y = 1.0109x
R² = 0.9139
MYH2
CKM
UQCRC2
SDHA
IDH3B
NDUFA8
Intensity Trial 2
Intensity Trial 1
d)
% Fibre Type
Type I pools
MYH7 MYH2 MYH1
e)
% Fibre Type
Type II Pools
MYH7 MYH2 MYH1
f)
Enrollment
Assessed for Eligibility (n = 28)
Randomised (n = 28)
Allocated to MICT (n=14)
Allocation
Allocated to SIT (n= 14)
Discontinued Intervention Due to Time Contraints (n=2)
Training Follow-Up
Discontinued Intervention Due to Time Contraints (n=2)
Analysed (n = 11) Excluded from analysis as sample was excessively fibrotic
Analysis
Analysed (n=12)
Analysed (n = 8) Excluded from analysis where the fibre typing with the dot blotting was inconsistent with the proteomics
Data Analysis
Analysed (n=8) Excluded from analysis where the fibre typing with the dot blotting was inconsistent with the proteomics
g)
MICT
SIT
Pre Training
Post Training
Type I
Type II
h)
1 2 3 4 5 6 7 8
A B C D E
MHC I
MHC IIa
i)
Group
Peptides
Proteins
Number of IDs
30000
20000
10000
0
4
8
12
16
Number of pooled Fibres

Supplementary Figure 1. Methodological approach to identify proteins in pools of single fibres.

a) The number of proteins quantified in the training study dataset by our proteomic workflow in all pooled single-fibre samples (n = 64). Samples are arranged according to TMT batches (A-H). Samples are labelled according to participant ID (Z to C), training type (S for SIT or M for MICT), biopsy time (PRE or POST training), and fibre type (I or II)).
b) Reproducibility of protein intensities (intensity-based absolute quantification; IBAQ) for replicates of type I skeletal muscle fibre samples. Core mitochondrial proteins (OXPHOS – NDUFA8, SDHA, URCRC2, COX5B, ATP5B, IDH3B and CKM) are highlighted in yellow, in addition to sarcomeric proteins (MYH 7 - blue and ACTA1 - orange).
c) Reproducibility of protein intensities (intensity-based absolute quantification; IBAQ) for replicates of type II skeletal muscle fibre samples. Core mitochondrial proteins (OXPHOS – NDUFA8, SDHA, URCRC2, COX5B, ATP5B, IDH3B and CKM) are highlighted in yellow, in addition to MYH2 (green).
d) Relative abundance of myosin heavy chains (MYH) in type I samples (n = 34) pooled by dot blotting, according to the percentage of the MYH7 (blue = type I), MYH2 (red = type-IIa), MYH1 (brown = type-IIx), and MYH4 (purple = type IIb) protein isoforms detected.
e) Relative abundance of myosin heavy chains (MYH) in type II samples (n = 36) pooled by dot blotting, according to the percentage of the MYH7 (blue = type I), MYH2 (red = type-IIa) MYH1 (brown = type-IIx), and MYH4 (purple = type IIb) protein isoforms detected.
f) Study recruitment flow chart and final group sizes used within the study.
g) Hierarchical visualisation of the statistical comparisons performed in this study.
h) Fibre typing by dot blotting. This example shows membrane images for the identification of fibre types in a single participant. Type I fibres were identified via the presence of MHCI (left panel), and type II fibres were identified via the presence of MHCIIa (right panel).
i) Depth of coverage in pooled single fibre samples (n = 6) for the number of proteins and peptides identified.

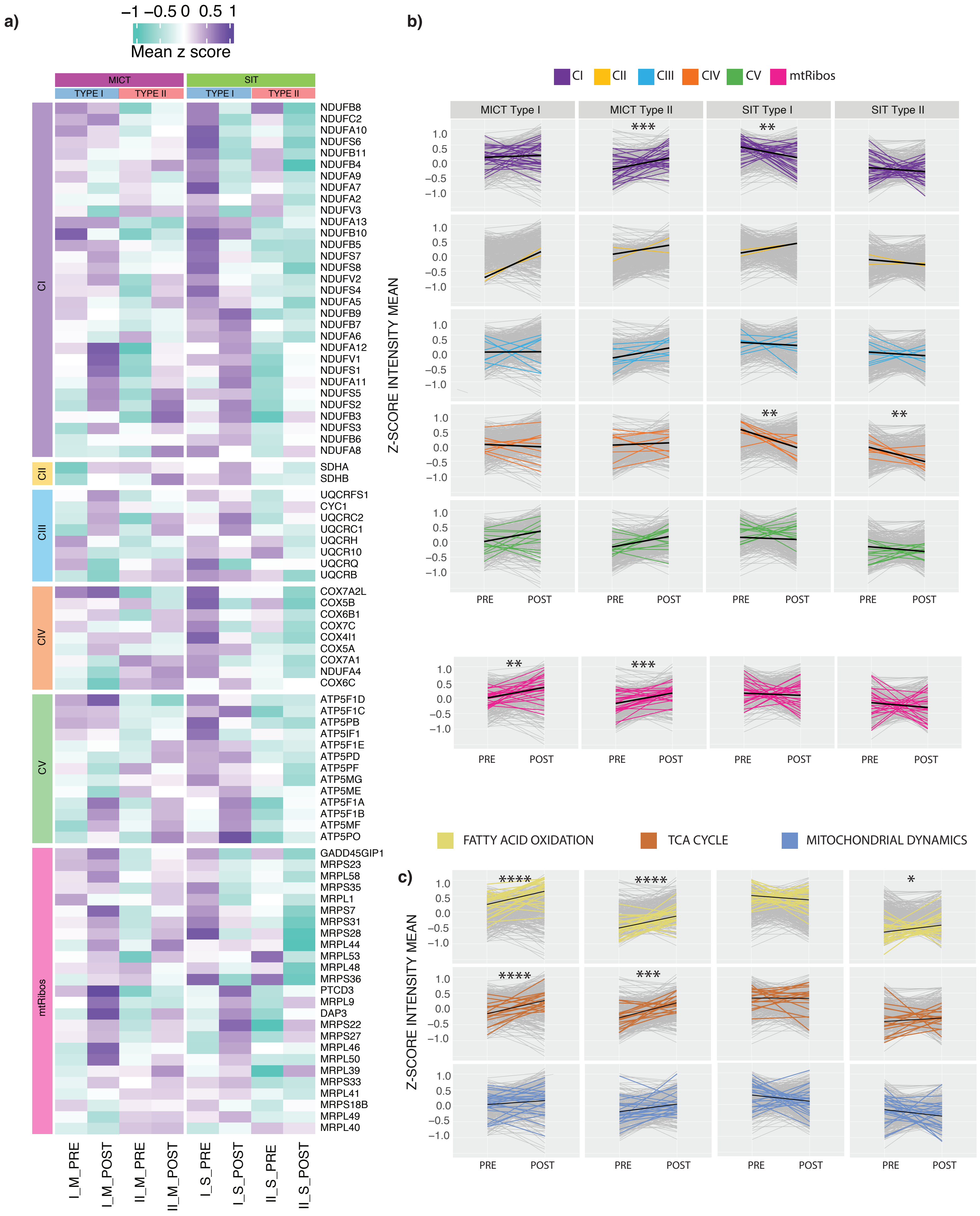

a)
−1 −0.5 0 0.5 1
Mean z score
MICT
SIT
TYPE I
TYPE II
TYPE I
TYPE II
CI
CII
CIII
CIV
CV
mtRibos
NDUFB8
NDUFC2
NDUFA10
NDUFS6
NDUFB11
NDUFB4
NDUFA9
NDUFA7
NDUFA2
NDUFV3
NDUFA13
NDUFB10
NDUFB5
NDUFS7
NDUFS8
NDUFV2
NDUFS4
NDUFA5
NDUFB9
NDUFB7
NDUFA6
NDUFA12
NDUFV1
NDUFS1
NDUFA11
NDUFS5
NDUFS2
NDUFB3
NDUFS3
NDUFB6
NDUFA8
SDHA
SDHB
UQCRFS1
CYC1
UQCRC2
UQCRC1
UQCRH
UQCR10
UQCRQ
UQCRB
COX7A2L
COX5B
COX6B1
COX7C
COX4I1
COX5A
COX7A1
NDUFA4
COX6C
ATP5F1D
ATP5F1C
ATP5PB
ATP5IF1
ATP5F1E
ATP5PD
ATP5PF
ATP5MG
ATP5ME
ATP5F1A
ATP5F1B
ATP5MF
ATP5PO
GADD45GIP1
MRPS23
MRPL58
MRPS35
MRPL1
MRPS7
MRPS31
MRPS28
MRPL44
MRPL53
MRPL48
MRPS36
PTCD3
MRPL9
DAP3
MRPS22
MRPS27
MRPL46
MRPL50
MRPL39
MRPS33
MRPL41
MRPS18B
MRPL49
MRPL40
I_M_PRE
I_M_POST
II_M_PRE
II_M_POST
I_S_PRE
I_S_POST
II_S_PRE
II_S_POST
b)
CI
CII
CIII
CIV
CV
mtRibos
MICT Type I
MICT Type II
SIT Type I
SIT Type II
Z-SCORE INTENSITY MEAN
1.0
0.5
0.0
−0.5
−1.0
***
**
**
**
**
***
PRE
POST
c)
FATTY ACID OXIDATION
TCA CYCLE
MITOCHONDRIAL DYNAMICS
Z-SCORE INTENSITY MEAN
****
****
*
****
***
PRE
POST

Supplemental Figure 2. Fibre-type differences in mitochondrial protein abundances on data not normalised for mitochondrial content.

- **a)** Heatmap displaying z-score values of protein subunits of the OXPHOS complexes, as well as the mitochondrial ribosome, in each of the two training groups, moderate-intensity continuous training (MICT) or sprint interval training (SIT), in type I and type II fibre type pools pre- and post-training, without the application of mitochondrial normalisation.
- **b)** Scaled profile plots without the application of mitochondrial normalisation showing changes in the relative abundance of the subunits in each of the oxidative phosphorylation (OXPHOS) complexes (CI to CV), as well as the mitochondrial ribosome, in type I and type II fibre types in response to both types of training (MICT and SIT). The mean of each group (all proteins identified in each pathway using MitoCarta 3.0) is indicated by the black line. Data are shown as Δ mean z-score. * indicates P value for significance (*: $P \leq 0.01$, **: $P \leq 0.001$, ***: $P \leq 0.0001$, ****: $P \leq 0.00001$) based on a paired t-test of the pre- to post-training values.
- **c)** Scaled profile plots without the application of mitochondrial normalisation showing changes in the relative abundance of proteins involved in fatty acid oxidation, the tricarboxylic acid cycle, and mitochondrial dynamics (all proteins identified in pathway using MitoCarta 3.0) in type I and type II fibre types in response to both types of training (MICT and SIT). The mean of each group is indicated by the black line. Data are shown as Δ mean z-score. * indicates P value for significance (*: $P \leq 0.01$, **: $P \leq 0.001$, ***: $P \leq 0.0001$, ****: $P \leq 0.00001$) based on a paired t-test of the pre- to post-training values.
