# Supplementary material for: Fibre-specific mitochondrial protein abundance is linked to resting and post-training mitochondrial content in the muscle of men
Source: Nat Commun. 2024 Sep 3;15:7677. doi: 10.1038/s41467-024-50632-2 (PMC11371815; doi:10.1038/s41467-024-50632-2)
Supplement: Supplementary file 3 — Description of Additional Supplementary Files [file 41467_2024_50632_MOESM3_ESM.pdf]

Description of Additional Supplementary Files

File name: Supplementary Data 1

Description: Participant characteristics, Raw intensities for all proteomic data, and log intensities for all identified proteins in all samples following the normalisation described in the methods. Data also includes gene ontology cellular compartment (GOCC) analysis for pooled single fibre analysis for percentage of cellular compartment identified (shown in Figure 1b) and the top 5 GOCC and corresponding $\log_{10}$ P value identified using the DAVID software for proteins identified by key single fibre proteomic studies (shown in Figure 1c). Further analysis includes the protein lists presented in the Venn diagram of Figure 1c for identified proteins within the current study, along with the studies of Deshmukh et al. and Murgia et al. (Tab 5), along with the top GOCC terms for the common proteins identified in all 3 studies (Figure 1d, Tab 6). Statistical analysis is lastly presented via logged intensities of proteins within the PRE type I and type II fibre samples. The table also displays calculations completed to calculate the 'Fusion Factor' used in Deshmukh et al. (2021) and the corresponding significance. Further detail is provided in the legend tab of the linked Excel file.

File name: Supplementary Data 2

Description: The list of mitochondrial proteins (according to Mitocarta 3.0) in common to all previous single fibre human studies pre intervention. Further analysis is presented in the table output by the DAVID software to identify top gene ontology biological processes (GOBP) groups of the mitochondrial proteins identified in over 70% of the total sample in all three studies listed (Deshmukh et al., Murgia et al., and the current study). Further analysis is presented with the average of all mitochondrial proteins in each individual. The mean of averages were calculated and compared between PRE training type I fibres and type II fibres. Mitochondrial protein expression (MPE) calculation is subsequently presented for identified mitochondrial proteins within the study in each comparison. Data normalised for mitochondrial content for identified mitochondrial proteins within the study for the PRE type I vs II comparison. Further detail provided in the legend tab of the linked Excel file.

File name: Supplementary Data 3

Description: Fold Difference from Pre to Post exercise for blood concentration of H^+ and lactate results. Further detail is provided for intensity and volume for each training group presented for analysis.

File name: Supplementary Data 4

Description: The normalised Data (Log2) with *limma* and Fusion Factor calculations for MICT Type I and Type II - PRE vs POST. In addition, Normalised Data (Log$_2$ with *limma* and Fusion Factor Calculations for SIT type I & type II - PRE vs POST. Parallel analysis is presented for PRE vs POST MICT type I for Deshmukh et al., 2021. Further detail is provided in the legend tab of the linked Excel file.

File name: Supplementary Data 5

Description: Log intensities for type I versus type II fibres in POST MICT and SIT samples following normalisation. GO Enrichment (Biological Processes) completed with DAVID on the common and unique MICT and SIT proteins identified when comparing Post I vs II samples. Further corresponding GOBP analysis is also made proteins identified as differentially expressed between type I and II fibres in the PRE samples. Further enrichment analysis is undertaken via WikiPathways enrichment analysis and Network analysis of differences in protein abundance between type I & type II fibres in the POST samples for MICT & SIT (analysed separately), using enrichplot & clusterprofiler on the *limma* results displayed in Tab 1 and 2 of this file. Further detail provided in the legend tab of the linked Excel file.

File name: Supplementary Data 6

Description: Differences from Pre to Post training for Mitochondria Volume Density (%) and Citrate Synthase activity (mol•h^{-1}•kg $protein^{-1}$) for MICT & SIT. Analysis presented for scaled intensities (z-scores) and means for known mitochondrial proteins for all samples following standard normalisation. Scaled means for each complex in each sample group are also presented in addition to the mean change for each comparison of training and fibre type. Further detail is provided in the legend tab of the linked Excel file.

File name: Supplementary Data 7

Description: Scaled intensities and means for known mitochondrial proteins for all samples following mitochondrial normalisation. Protein functional classes and metabolic pathways presented with the scaled means (z-scores) for each functional group (defined by MitoCarta 3.0) in each sample group in addition to the mean change for each comparison of training and fibre type with mitochondrial normalisation applied. Further detail provided in the legend tab of the linked Excel file.

File name: Supplementary Data 8

Description: Protein functional classes and metabolic pathways presented with the scaled means (z-scores) for each functional group (defined by MitoCarta 3.0) in each sample group in addition to the mean change for each comparison of training and fibre type. Further detail is provided in the legend tab of the linked Excel file.

File name: Supplementary Data 9

Description: The number of proteins quantified in the training study dataset by our proteomic workflow in all pooled single-fibre samples. Raw TMT Batch ID is presented and arranged according to TMT batches (A-H). Samples are labelled according to participant ID (Z-C), training type (S for SIT or M for MICT), training time (pre or post) and fibre type (I or II). In addition, reliability between replicates is presented - List of Intensity reported by intensity-based absolute quantification (IBAQ) for replicates and corresponding gene names of pooled type I fibres (n = 6 fibres) and type II fibres. Furthermore, the relative TMT intensity and percentage abundance of myosin heavy chains (MYH) for type I and type II samples pooled by dot blotting is presented.
