# Supplementary material for: Fibre-specific mitochondrial protein abundance is linked to resting and post-training mitochondrial content in the muscle of men
Source: Nat Commun. 2024 Sep 3;15:7677. doi: 10.1038/s41467-024-50632-2 (PMC11371815; doi:10.1038/s41467-024-50632-2)
Supplement: Supplementary file 13 — Reporting Summary [file 41467_2024_50632_MOESM13_ESM.pdf]

nature portfolio

Corresponding author(s): Prof. David Bishop, Dr. Nikeisha Caruana

Last updated by author(s): Nov 16, 2023

Reporting Summary

Nature Portfolio wishes to improve the reproducibility of the work that we publish. This form provides structure for consistency and transparency in reporting. For further information on Nature Portfolio policies, see our Editorial Policies and the Editorial Policy Checklist.

Statistics

For all statistical analyses, confirm that the following items are present in the figure legend, table legend, main text, or Methods section.

n/a	Confirmed	
☐	☒	The exact sample size (*n*) for each experimental group/condition, given as a discrete number and unit of measurement
☐	☒	A statement on whether measurements were taken from distinct samples or whether the same sample was measured repeatedly
☐	☒	The statistical test(s) used AND whether they are one- or two-sided<br>*Only common tests should be described solely by name; describe more complex techniques in the Methods section.*
☒	☐	A description of all covariates tested
☐	☒	A description of any assumptions or corrections, such as tests of normality and adjustment for multiple comparisons
☐	☒	A full description of the statistical parameters including central tendency (e.g. means) or other basic estimates (e.g. regression coefficient) AND variation (e.g. standard deviation) or associated estimates of uncertainty (e.g. confidence intervals)
☐	☒	For null hypothesis testing, the test statistic (e.g. *F*, *t*, *r*) with confidence intervals, effect sizes, degrees of freedom and *P* value noted<br>*Give P values as exact values whenever suitable.*
☒	☐	For Bayesian analysis, information on the choice of priors and Markov chain Monte Carlo settings
☒	☐	For hierarchical and complex designs, identification of the appropriate level for tests and full reporting of outcomes
☒	☐	Estimates of effect sizes (e.g. Cohen's *d*, Pearson's *r*), indicating how they were calculated

Our web collection on statistics for biologists contains articles on many of the points above.

Software and code

Policy information about availability of computer code

Data collection	R v3.6.3; packages: Limma (v3.48.3) ,impute (v1.76.0), Enrichr (v3.2), ComplexHeatmap package (v.2.8.0), VennDiagram (v1.7.1), ClusterProfiler (v4.0.5). Adobe Illustrator(CC2018.22.1), R scripts detailed in the code availability section available at https://doi.org/10.5281/zenodo.7227800. ImageLab 5.2.1, BioRad. Cell F software (Olympus). Image J software (National Institutes of Health, Maryland, USA). DigitalMicrograph (Version 1.71.38) acquisition software.
Data analysis	Proteome Discoverer 2.4 (ThermoFisher) and the non-normalised protein reporter intensity was exported to Excel and further analysed in R

For manuscripts utilizing custom algorithms or software that are central to the research but not yet described in published literature, software must be made available to editors and reviewers. We strongly encourage code deposition in a community repository (e.g. GitHub). See the Nature Portfolio guidelines for submitting code & software for further information.

Data

Policy information about availability of data

All manuscripts must include a data availability statement. This statement should provide the following information, where applicable:

- Accession codes, unique identifiers, or web links for publicly available datasets
- A description of any restrictions on data availability
- For clinical datasets or third party data, please ensure that the statement adheres to our policy

Source data to interpret, verify, and extend this research is provided with this paper. The mass spectrometry proteomics data has been deposited in the ProteomeXchange Consortium via the PRIDE partner repository under accession code PXD036010.

Research involving human participants, their data, or biological material

Policy information about studies with human participants or human data. See also policy information about sex, gender (identity/presentation), and sexual orientation and race, ethnicity and racism.

Reporting on sex and gender	Male participants were included, comparisons to gender and/or sex were not considered in this study design
Reporting on race, ethnicity, or other socially relevant groupings	Individuals self-reported for sex
Population characteristics	Sixteen young, healthy, male, participants (27.5 ± 5.2 y; 177 ± 7 cm; 73.8 ± 8.7 kg; 23.6 ± 2.6 BMI) were included in the final analysis.
Recruitment	Participants were recruited from the Melbourne area in Victoria, Australia, by advertising on the University campus noticeboards and the surrounding areas (Footscray), and by presenting the study before University classes without bias toward a particular department or faculty (Footscray Park Campus). Other than the above, self selection bias was not controlled; however, we believe the impact to be minimal as the above population characteristics for untrained individuals were applied. The participants gave their written, informed consent to participate in the study. Approval for the study procedures, which confirmed to the standards set by the latest revision of the Declaration of Helsinki, was granted by the Victoria University Research Ethics Committee (HRE17-075). Recruitment was based on the following inclusion and exclusion criteria: Inclusion criteria were:<br><br>Key inclusion criteria<br>Active participants (taking part in physical activity 1 to 3 times per week)<br>Free of injury<br>Free of any adverse health condition.<br><br>Minimum age<br>18 Years<br><br>Maximum age<br>35 Years<br><br>Gender<br>Males<br><br>Key exclusion criteria<br>Any of the following:<br>1) sedentary<br>2) current muscle or ligament injury of the lower body.<br>3) current or previous cardiovascular or respiratory condition or abnormality.<br>4) current metabolic disease (e.g. diabetes)
Ethics oversight	Victoria University Human Research Ethics Committee (HRE17-075)

Note that full information on the approval of the study protocol must also be provided in the manuscript.

Field-specific reporting

Please select the one below that is the best fit for your research. If you are not sure, read the appropriate sections before making your selection.

☒ Life sciences ☐ Behavioural & social sciences ☐ Ecological, evolutionary & environmental sciences

For a reference copy of the document with all sections, see nature.com/documents/nr-reporting-summary-flat.pdf

Life sciences study design

All studies must disclose on these points even when the disclosure is negative.

Sample size	The sample size (n) required to reach a significant level of 0.05 with a sufficient power of 80% was calculated for the following variables: mitochondrial volume, mitochondrial cristae density and mitochondrial respiration. This was based on studies with a lower training volume or duration than the present study, and previous research has shown that differences between groups are likely. Based on this a sample size of 8-14 per group was suggested as sufficient to reach the level of significance required for the overarching study. Twenty-eight healthy men initially volunteered to take part in this study. The data from sixteen participants (27.5 ± 5.2 y; 177 ± 7 cm; 73.8 ± 8.7 kg; 23.6 ± 2.6 BMI; Table S1 – Tab 1) was included in the final analysis. For proteomic analysis a sample size of 5 or greater is sufficient to provide detailed proteome adaptation in skeletal muscle (Schönke et al Proteomics, 2018)
Data exclusions	Two participants from the MICT group and two from the SIT group withdrew from the study due to time constraints, and the data from one participant was excluded from the final analysis as their muscle samples were of poor quality. Samples were also excluded where fibre typing by dot blotting was inconsistent with the proteomic data for a final sample size of n = 16 (see study recruitment flow chart and final group sizes; Figure S2f).
Replication	Due to the limited availability of biopsy material and the use of this material to conduct multiple LCMS experiments, as well as the limitations on the LCMS instrumentation time, all experiments were performed only once for the 16 subjects for PRE and POST conditions (including each training type and fibre type) . We verified the reproducibility of all analyses using a multidimensional scaling analysis and biological replicates (MDS) as well the number of peptide and protein identifications for each replicate. The attempts that were used for checking the reproducibility this way were successful.
Randomization	Randomization was applied for the study.
Blinding	Yes, the investigators were blinded to the group.

Reporting for specific materials, systems and methods

We require information from authors about some types of materials, experimental systems and methods used in many studies. Here, indicate whether each material, system or method listed is relevant to your study. If you are not sure if a list item applies to your research, read the appropriate section before selecting a response.

Materials & experimental systems

n/a	Involved in the study	
☐	☒	Antibodies
☒	☐	Eukaryotic cell lines
☒	☐	Palaeontology and archaeology
☒	☐	Animals and other organisms
☐	☒	Clinical data
☒	☐	Dual use research of concern
☒	☐	Plants

Methods

n/a	Involved in the study	
☒	☐	ChIP-seq
☒	☐	Flow cytometry
☒	☐	MRI-based neuroimaging

Antibodies

Antibodies used	Myosin heavy chain Type IIA, Developmental Studies Hybridoma Bank (DSHB), A4-74, RRID: AB_528383 diluted 1:200<br>Anti-Myosin heavy chain Type IIB, Developmental Studies Hybridoma Bank (DSHB) A4-840; RRID: AB_528384 diluted 1:200<br>Goat anti-Mouse IgG (H+L) Highly Cross-Adsorbed Secondary Antibody, Alexa Fluor Plus 488, ThermoFisher Scientific A32723; RRID: AB_2633275 diluted 1:20000<br>Goat anti-Mouse IgM (Heavy chain) Cross-Adsorbed Secondary Antibody, Alexa Fluor 488, ThermoFisher Scientific A-21042; RRID: AB_2535711 diluted 1:20000<br>Myosin heavy chain (slow, alpha- and beta-), Developmental Studies Hybridoma Bank (DSHB) BA-F8, RRID: AB_10572253 diluted 1:25<br>Myosin heavy chain (all but 2X) antibody, Developmental Studies Hybridoma Bank (DSHB) BF-35, RRID: AB_2274680 diluted 1:25<br>Goat anti-Mouse IgG2b Cross-Adsorbed Secondary Antibody, Alexa Fluor 350, ThermoFisher Scientific A-21140, RRID: AB_2535777 diluted 1:500<br>Goat anti-Mouse IgG1 Cross-Adsorbed Secondary Antibody, Alexa Fluor 488, ThermoFisher Scientific A-21121, RRID: AB_2535764 diluted 1:500
Validation	Myosin heavy chain Type IIA, Developmental Studies Hybridoma Bank (DSHB), A4-74, RRID: AB_528383<br>The manufacturer has 36 citations for this antibody on their<br>website:<br>https://dshb.biology.uiowa.edu/A4-74<br>First published with expected results: by Webster et al. DOI: 10.1016/0092-8674(88)90463-1<br><br>Anti-Myosin heavy chain Type IIB, Developmental Studies Hybridoma Bank (DSHB) A4-840; RRID: AB_528384<br>The manufacturer has 45 citations for this antibody on their

website:
https://dshb.biology.uiowa.edu/A4-840
First published with expected results: by Webster et al. DOI: 10.1016/0092-8674(88)90463-1

Goat anti-Mouse IgG (H+L) Highly Cross-Adsorbed Secondary Antibody, Alexa Fluor Plus 488, ThermoFisher Scientific A32723; RRID: AB_2633275
The manufacturer has 1015 citations for this antibody on their
website:
https://www.thermofisher.com/antibody/product/Goat-anti-Mouse-IgG-H-L-Highly-Cross-Adsorbed-Secondary-Antibody-Polyclonal/A32723
The manufacturer has validated this antibody using imaging to show the specificity of the secondary antibody in multiple cell types

Goat anti-Mouse IgM (Heavy chain) Cross-Adsorbed Secondary Antibody, Alexa Fluor 488, ThermoFisher Scientific A-21042; RRID: AB_2535711
The manufacturer has 441 citations for this antibody on their
website:
https://www.thermofisher.com/antibody/product/Goat-anti-Mouse-IgM-Heavy-chain-Cross-Adsorbed-Secondary-Antibody-Polyclonal/A-21042
The manufacturer has validated this antibody using imaging to show the specificity of the secondary antibody in multiple cell types

Myosin heavy chain (slow, alpha- and beta-), Developmental Studies Hybridoma Bank (DSHB) BA-F8, RRID: AB_10572253
The manufacturer has 38 citations for this antibody on their
website:
https://dshb.biology.uiowa.edu/BA-F8
First published with expected results: by Barrione et al. DOI: 10.1111/j.1432-0436.1988.tb00591.x.

Myosin heavy chain (all but 2X) antibody, Developmental Studies Hybridoma Bank (DSHB) BF-35, RRID: AB_2274680
The manufacturer has 20 citations for this antibody on their
website:
https://dshb.biology.uiowa.edu/BF-35
First published with expected results by Schiaffino et al. DOI: 10.1007/BF01739810.

Goat anti-Mouse IgG2b Cross-Adsorbed Secondary Antibody, Alexa Fluor 350, ThermoFisher Scientific A-21140, RRID: AB_2535777
The manufacturer has 87 citations for this antibody on their
website:
https://www.thermofisher.com/antibody/product/Goat-anti-Mouse-IgG2b-Cross-Adsorbed-Secondary-Antibody-Polyclonal/A-21140
The manufacturer has validated this antibody using imaging to show the specificity of the secondary antibody in multiple cell types

Goat anti-Mouse IgG1 Cross-Adsorbed Secondary Antibody, Alexa Fluor 488, ThermoFisher Scientific A-21121, RRID: AB_2535764
The manufacturer has 1011 citations for this antibody on their
website:
https://www.thermofisher.com/antibody/product/Goat-anti-Mouse-IgG1-Cross-Adsorbed-Secondary-Antibody-Polyclonal/A-21121
The manufacturer has validated this antibody using imaging to show the specificity of the secondary antibody in multiple cell types

Clinical data

Policy information about clinical studies
All manuscripts should comply with the ICMJE guidelines for publication of clinical research and a completed CONSORT checklist must be included with all submissions.

Clinical trial registration: Australian New Zealand Clinical Trials Registry (ANZCTR; ACTRN12617001105336).

Study protocol: Full intervention protocol can be viewed on the Australian New Zealand Clinical Trials Registry website under trial number ACTRN12617001105336 or at https://www.anzctr.org.au/Trial/Registration/TrialReview.aspx?id=373202&isReview=true

Data collection: Date of last data collection
Anticipated 30/06/2019 Actual 24/12/2018

Outcomes: Primary outcome [1] Mitochondrial content measured as mitochondrial volume are from electron microscopy (EM) images and citrate synthase activity from the enzyme activity assay. Both measurements are from skeletal muscle obtained from the vastus lateralis.
Timepoint [1] Before the first exercise session in week 1 and 72h after the last exercise session in week 8.
Primary outcome [2] Mitochondrial respiratory function in permeabilised fibres assessed with an oxygen respirometer that tracks changes in oxygen levels when substrates are added to the permeabilised fibres..
Timepoint [2] Before the first exercise session in week 1 and 72h after the last exercise session in week 8.
Primary outcome [3] Mitochondrial dynamics measured as protein content (via Western Blot technique) and gene expression (via quantitative polymerase chain reaction technique; qPCR) of MFN2, and OPA1 (mitochondrial fusion); protein content and gene expression of DRP1 and Mff (mitochondrial fission). All measurements are from skeletal muscle obtained from the vastus lateralis.This is a composite primary outcome.
Timepoint [3] Before and after the first exercise session in week 1.
Secondary outcome [1] Relationship between endurance performance adaptation (measured as time to complete 20 km) and mitochondrial content (as citrate synthase activity from the enzyme activity assay, and mitochondrial volume density from electron microscopy images from the skeletal muscle samples obtained from the vastus lateralis).

Timepoint [1] Before the first exercise session in week 1 and 72h after the last exercise session in week 8.
Secondary outcome [2] Mitochondrial specific degradation (mitophagy) measured as protein content (via Western Blot technique) and gene expression (via quantitative polymerase chain reaction technique; qPCR) of markers such as PINK1, phosphorylation of Ubiquitin at Serine 65, and Parkin. All measurements are from skeletal muscle obtained from the vastus lateralis. This is a composite secondary outcome.
Timepoint [2] Before and after the first exercise session in week 1.

Plants

Seed stocks

Report on the source of all seed stocks or other plant material used. If applicable, state the seed stock centre and catalogue number. If plant specimens were collected from the field, describe the collection location, date and sampling procedures.

Novel plant genotypes

Describe the methods by which all novel plant genotypes were produced. This includes those generated by transgenic approaches, gene editing, chemical/radiation-based mutagenesis and hybridization. For transgenic lines, describe the transformation method, the number of independent lines analyzed and the generation upon which experiments were performed. For gene-edited lines, describe the editor used, the endogenous sequence targeted for editing, the targeting guide RNA sequence (if applicable) and how the editor was applied.

Authentication

Describe any authentication procedures for each seed stock used or novel genotype generated. Describe any experiments used to assess the effect of a mutation and, where applicable, how potential secondary effects (e.g. second site T-DNA insertions, mosiacism, off-target gene editing) were examined.
