# Supplementary figures and images for: Fibre-specific mitochondrial protein abundance is linked to resting and post-training mitochondrial content in the muscle of men
Source: Nat Commun. 2024 Sep 3;15:7677. doi: 10.1038/s41467-024-50632-2 (PMC11371815; doi:10.1038/s41467-024-50632-2)

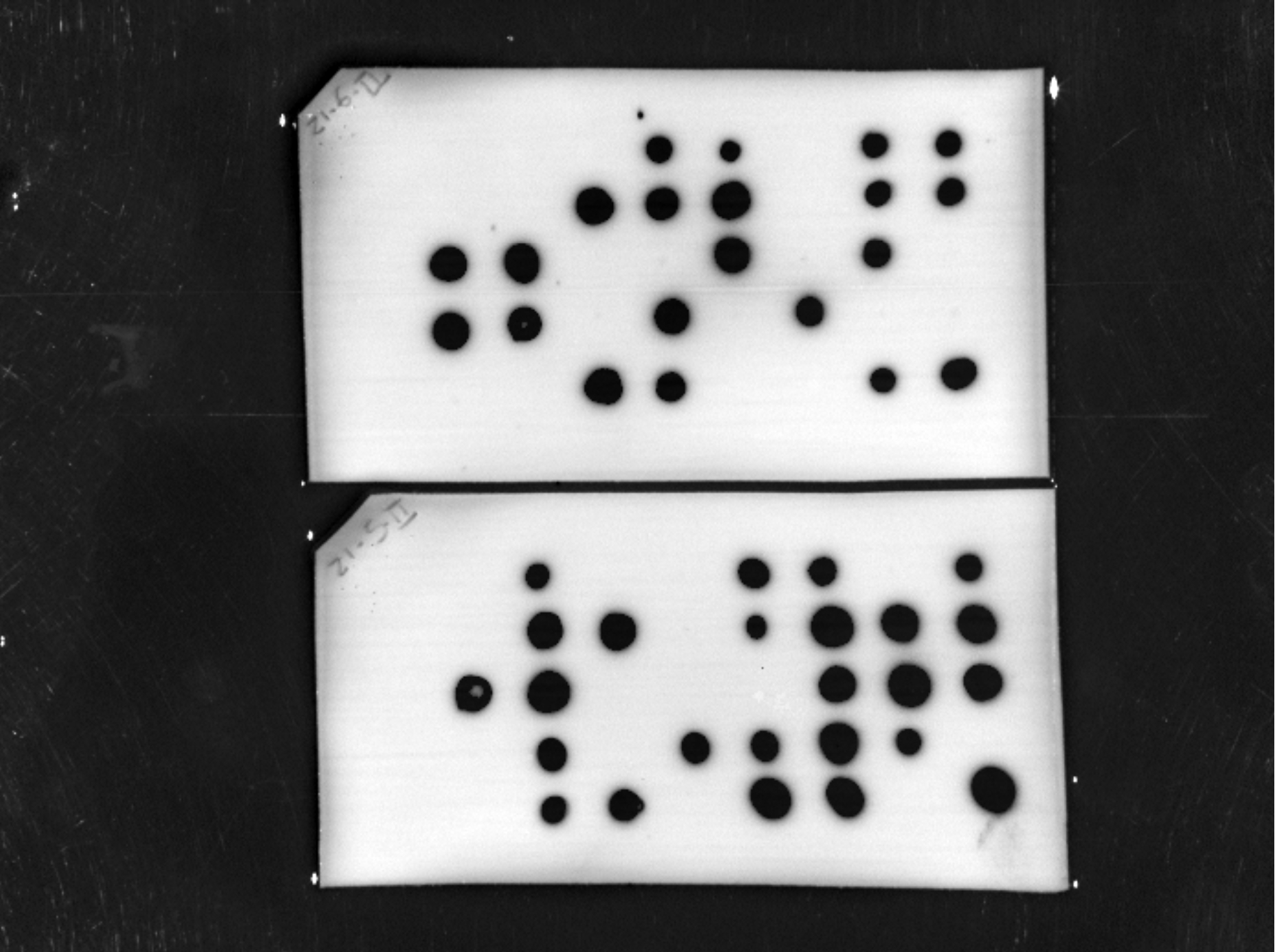

Supplement: Supplementary file 14 — Source Data [file 41467_2024_50632_MOESM14_ESM.zip › SourceData/21-6-II_21-5-II.jpg]

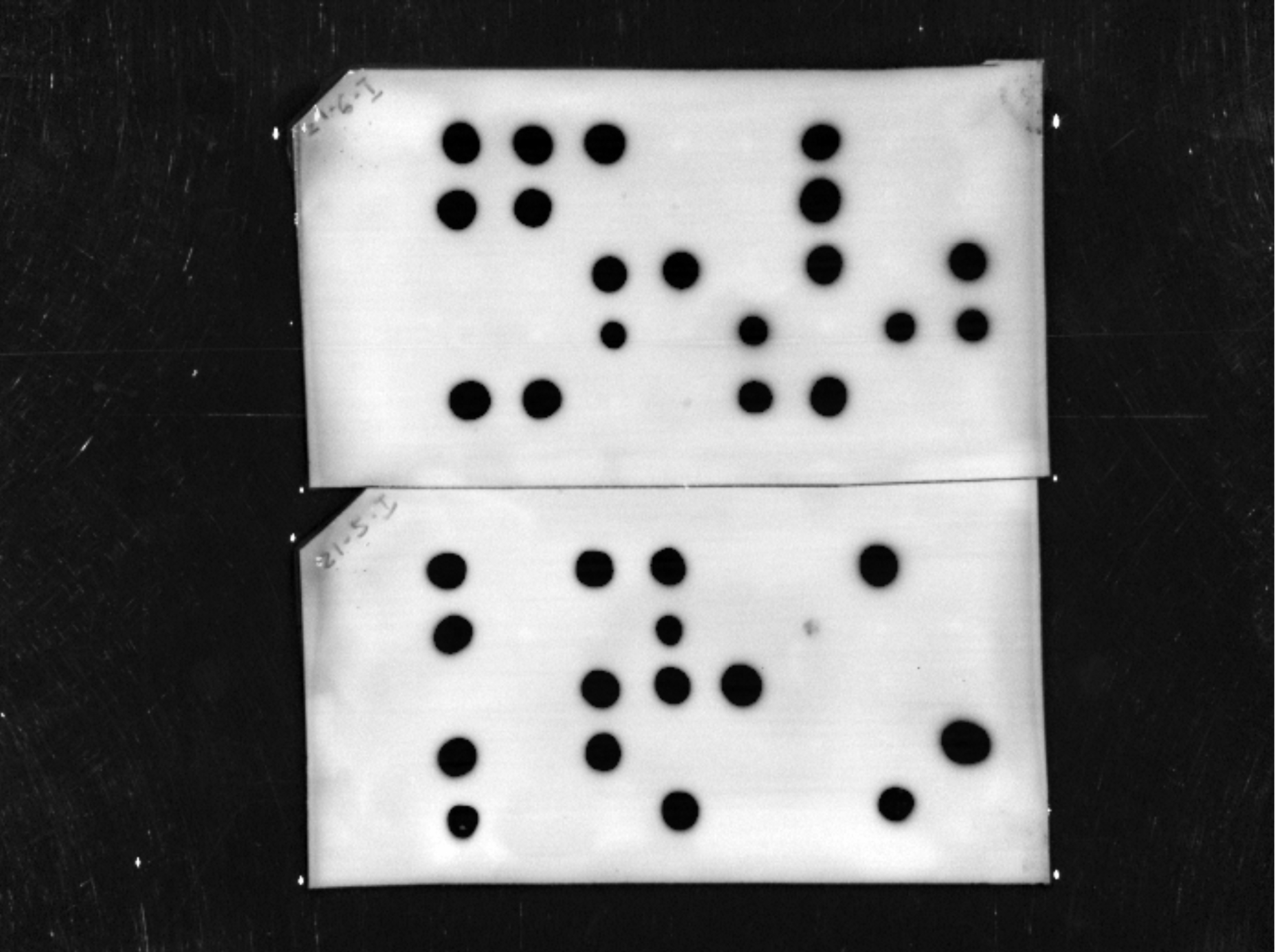

Supplement: Supplementary file 14 — Source Data [file 41467_2024_50632_MOESM14_ESM.zip › SourceData/21-6-I_21-5-I.jpg]
